# Youheng Tang, the pioneering founder of South China Agricultural University

**DOI:** 10.1007/s13238-020-00815-6

**Published:** 2021-01-12

**Authors:** Yuan Chen, Alan W. Adame, Ge-Zhi Chen, Yuanyuan Meng

**Affiliations:** 1 South China Agricultural University, Guangzhou, 510642 China; 2 Shenzhen Middle School International Department, Shenzhen, 518001 China; 3 Beijing Institutes of Life Science, Chinese Academy of Sciences, Beijing, 100101 China

Living during the eve of the Qing Dynasty, Youheng Tang (唐有恒) was a pioneer of agricultural education and agronomy research in China. He sought neither money nor vanity, only to fulfill his deep-seated dream to benefit the nation. In addition to being a Chinese agricultural educator, agricultural sciences scholar, and modern agricultural science and education entrepreneur in Guangdong Province, Youheng Tang was also the founder of South China Agricultural University (Fig. 1).

**Figure 1:**
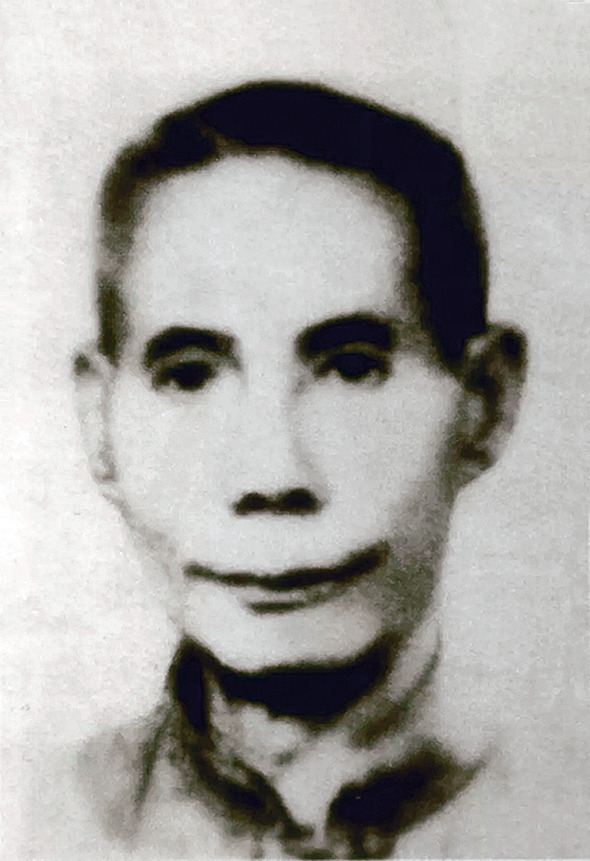
Dr. Youheng Tang, the founder of Guangdong Provincial Agricultural Testing Ground, graduated from Cornell University’s Agricultural College

## VOWING TO SERVE THE COUNTRY WITH DILIGENCE AND HARD WORK

On May 7th, 1885, Youheng Tang was born to a peasant family in Tangjia village, Xiangshan County (now located in Tangjia village, Zhuhai). From birth, “agriculture” was in his blood and would be bound with him throughout his life.

At age 16, Youheng Tang following his brother went to Queen’s College in Hong Kong to study English. In 1904, at the age of 19, Youheng Tang passed the official exams, and was sponsored by the Qing Dynasty to study in the United States. He studied physical chemistry at the University of California-Berkeley, and then went on to study agriculture at Cornell University’s Agricultural College, extensively researching a variety of rice breeding methods. When he was 22, Youheng Tang received a doctorate degree in agriculture from Cornell. Shortly after, he was hired as a technician at the U.S. Federal Ministry of Agriculture and became an honorary member of the Science Society.

During his studies in the United States, Youheng Tang acutely remained aware of China’s situation of gradual, social and political decline. After the defeat of the Sino-Japanese War, the Qing Dynasty ceded and gave up part of territorial ownership in order to pay the war reparations, which was a great humiliation to China. In those days, China was troubled by domestic strife and foreign invasion, swaging in the midst of the raging storm, and various unequal treaties simultaneously rocked China. At the time, although studying in the United States, Youheng Tang was determined to come back and save his home country. Reflecting on China’s turbulent situation and his own responsibility to his motherland, Youheng Tang once wrote: “There is a need to be honed, which is to repay the country with supreme loyalty; there is no time to be idle when there is an obvious need to bring up the nation.” In 1908, Youheng Tang, 23 years old, embraced his heart-felt duty to serve his country. He resolutely gave up the generous offers, benefits, and employment in the United States, and embarked on his journey back to China.

**Figure 2:**
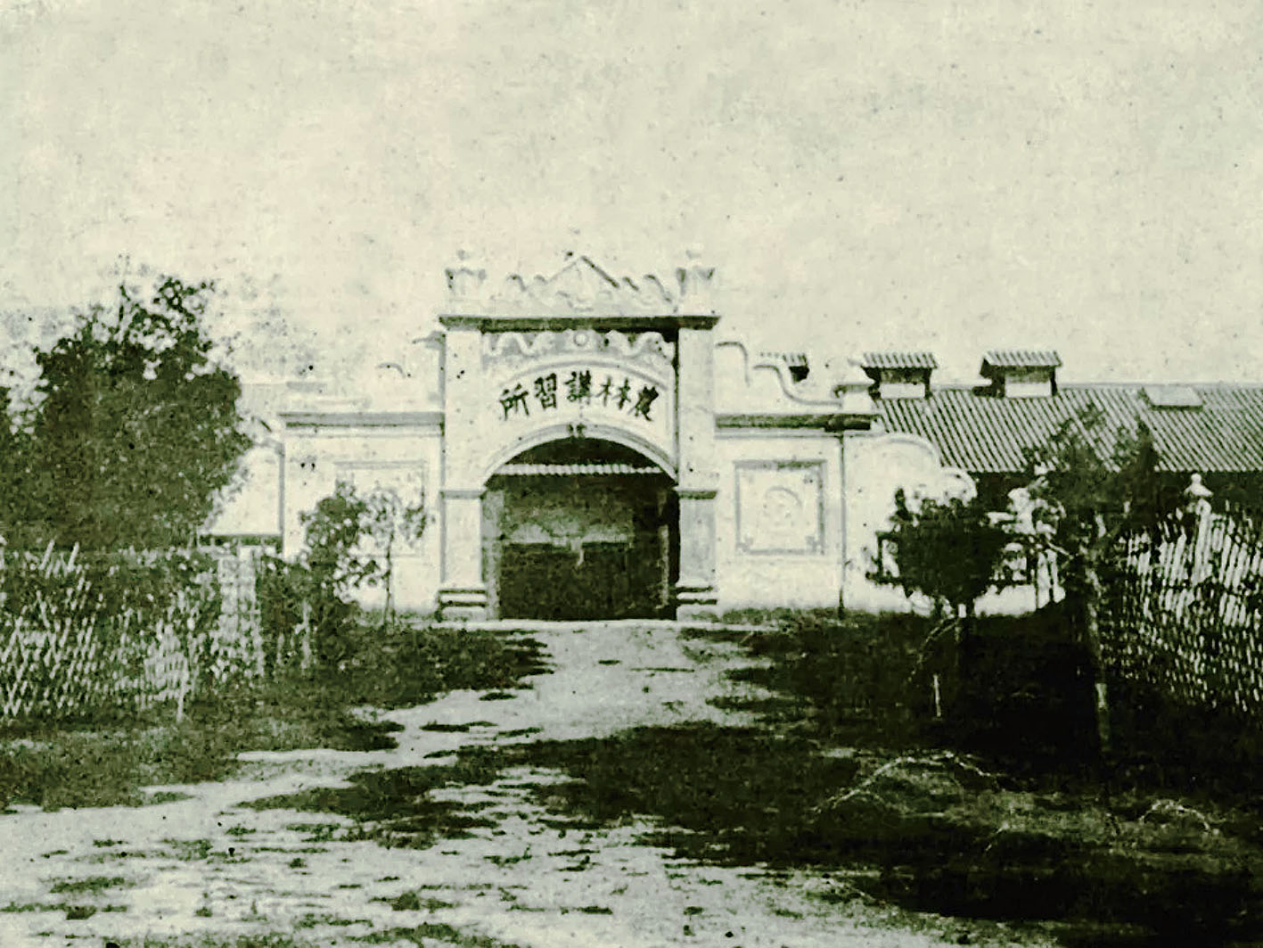
The Agricultural Workshop attached to Guangdong Provincial Agricultural Testing Ground

After the failure of the Westernization Movement in China, people with insight vigorously advocated for “saving the country by industry” and “saving the country by education”. In the 23th year of GuangXu during the Qing Dynasty (1897), government officials around the country issued new regulations to modernize Chinese agriculture, starting from the translation of many foreign agricultural books. After the birth of the “New Governance” in 1908, Guangdong Province established the “Quanye” Official department, which was responsible for the affairs of agriculture, industry and business. Youheng Tang was recommended by Mr. Wangzeng Chen (陈望曾), a local government official who had been keeping track of his achievements, to be appointed as the agriculture professor of Guangdong and Guangxi Province. Tang was in charge of planning and preparing the establishment of agricultural testing site at the Guangdong Provincial Agricultural Testing Ground. He chose and purchased the land, oversaw every single thing personally, actualizing the simultaneous construction of the factory and school, as well as recruiting talented and high-quality teachers. In 1909, the first year of the reign of the last king of the Qing Dynasty, Youheng Tang completed all preparations for the new agricultural institute and began recruiting students (Fig. 2).

## EXPLORING WAYS OF FEEDING A NATION IN YEARS OF HARDSHIP

Youheng Tang was well aware that government support and recognition can bring great long-term success to his school. In 1910, after excellent performance in his studies abroad, Youheng Tang passed the government official examination organized by the Qing government. He successfully passed the highest imperial exam, and from then on he was known as “Agricultural Scholar”, and soon thereafter the Qing government conferred him with a position at the Imperial Academy. Youheng Tang was a member of the last batch of imperially accredited scholars under Chinese feudal society while also having served as a member of a foreign national academy.

**Figure 3:**
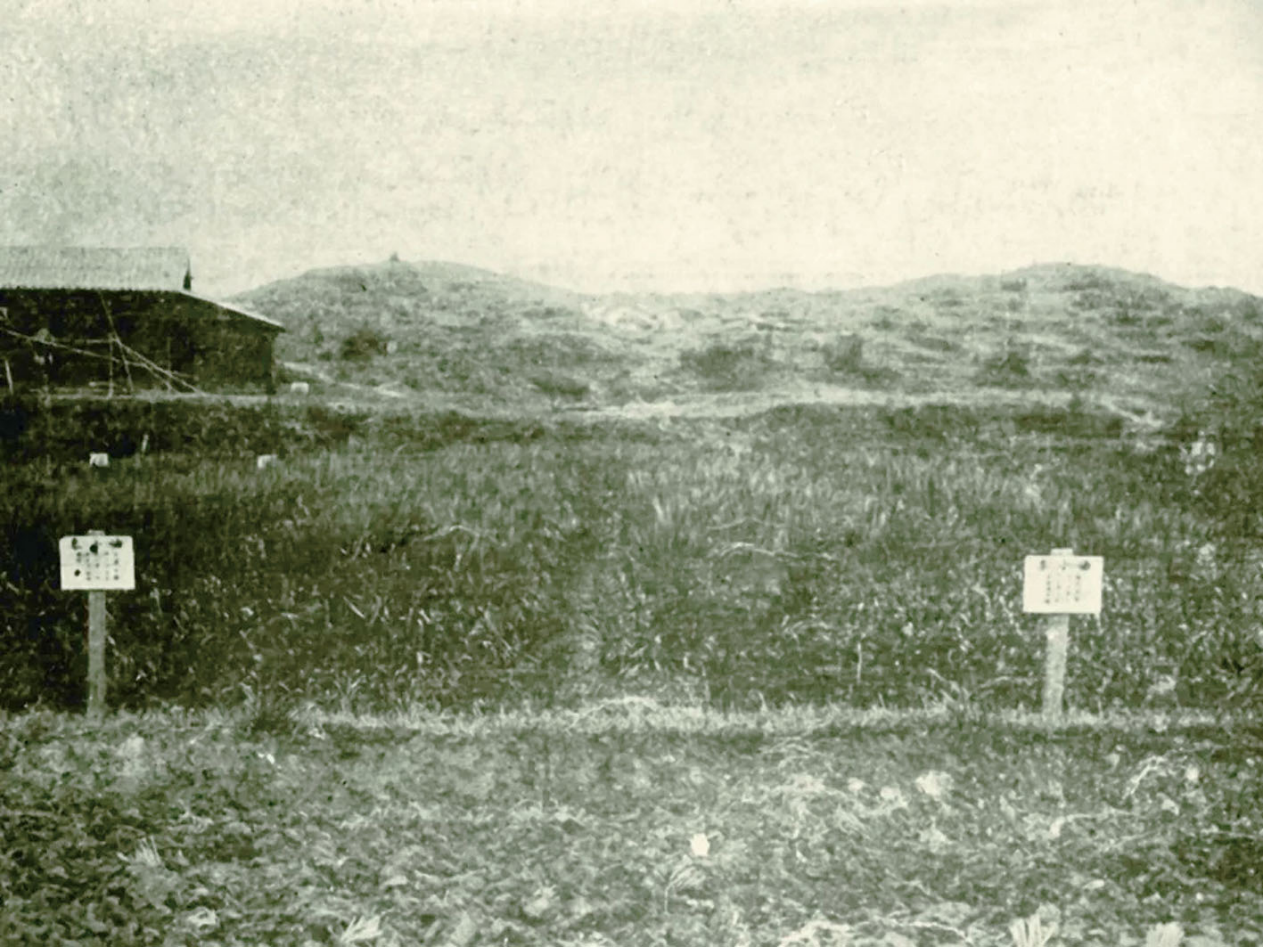
Agricultural Testing Ground

A series of appointments and honors marked the beginning of Youheng Tang’s official public career, but he had no intention of remaining within the bureaucratic circle. Fame and fortune did not give him any pleasure, so that he eventually gave up his official position at the Imperial Academy and returned to Guangdong province to continue devoting his time to the construction of the agricultural teaching-class. Through his tireless and unremitting efforts, in just a few years, the Guangdong Provincial Agricultural Testing Ground and its attached institutes were expanded into Guangdong Public Agricultural Specialized School, which was a significant infrastructural development in China’s modern agricultural education capability (Fig. 3).

In 1911, Youheng Tang continued his dedication to improve China’s agricultural education, adopting a “do-it-yourself, down-to-earth” approach to everything he did. After the overthrow of the Qing Dynasty in the Revolution of 1911, Youheng Tang would once again have to put his development of agricultural testing site and school on hold, and was dispatched to Hunan and Anhui by the government of the Republic of China to investigate the worsening flood situation. After field investigations and careful study, Youheng Tang proposed a targeted plan to prevent local disaster. In 1918, five north provinces in China suffered from drought and famine. Youheng Tang was once more involved in the rescue. He organized the “Huayang Donation Society” and held an “underground shafting training course”, providing his own strength for resistance to local disaster. However, as he had spent most of his time on the front line of disaster areas during these years, the strenuous physical effort began to take a toll on his body, and he became ill with hemoptysis.

Over the next few years after recovering from his illness, Youheng Tang vigorously promoted livestock breeding and running farms from north to south, leaving his footprints in Hebei province, Shandong province, Jiangsu province, and Zhejiang province. Youheng Tang applied his farming knowledge to the curriculum at the Agricultural Test Field in Guangzhou. He incorporated agronomic techniques that he learned from his studying in the United States into his research and future development, producing precocious corn seed, long cashmere cotton seed, and smut resistant wheat seed. These techniques not only improved productivity, but also cultivated other high-quality varieties. Tang actively improved farming tools: his revamped ploughshare and corn thresher, greatly improving the efficiency of farmers nationwide. At the National Agricultural Technology Exhibition, Youheng Tang’s work, Promoting Excellent Seed Touring Vehicles, was supported by railway authorities. This work was widely publicized in rural areas along the railway to let farmers understand the new knowledge and agricultural technology. Wherever he worked around the country, Youheng Tang greatly emphasized the importance of education. In addition to providing extensive training to farmers for greatly enhancing the capability in specialized agriculture and farming, he also set up eight schools for children of farmers across the different provinces where he worked.

With a deep understanding of Chinese agronomy, Youheng Tang’s well thought-out ambitions for China’s agricultural revolution led him to provide many illuminating insights for the development of Chinese agriculture and forestry. During his tenure as the secretary-general of Zhongshan County, Youheng Tang conducted a set of investigations about the agricultural and forest situation in the area, and wrote four articles, one of which was “An Outline of Zhongshan Model County Agriculture and Forestry Construction”. The content of his profound writing promoted the primary establishment of a “National Agricultural Model Village” at the junction between Zhongshan and Zhuhai. With the foresight and ambition to revive his hometown, Youheng Tang, an agricultural scientist, sketched a grand blueprint for the development and progress of agriculture across the country, intending to leverage the pioneering advances of agriculture and forestry development in Zhongshan County. Unfortunately, due to the social turmoil at that time, he fell short of achieving this grand vision.

## STRIVING TO CULTIVATE EXCELLENT STUDENTS FOR THE COUNTRY

Guangdong Provincial Agricultural Testing Ground and its attached agricultural testing site were early predecessors to South China Agricultural University and were also incipient products of Youheng Tang’s painstaking efforts. In 1908, Youheng Tang was appointed as the director and chief agronomist. After being appointed, he immediately surveyed the east side of Xiniupian in front of Our Village, Dongmenwai (present-day Ouzhuang District) in Guangzhou, and decided to use this area as the education site. Concurrently, he purchased 98 acres of land as a site for fieldwork and experiments. A two-story teaching building was built in the Shimagang area of Dongshan. Later that same year, a Chinese-Indonesian, Zhenxun Zhang (张振勋), donated 80 acres of land, increasing the total field site area to 178 acres. In October 1908, preparation office was established on the testing site, which meant that there were added classrooms, laboratories, a library, an auditorium, and a dining hall in the new school.

**Figure 4:**
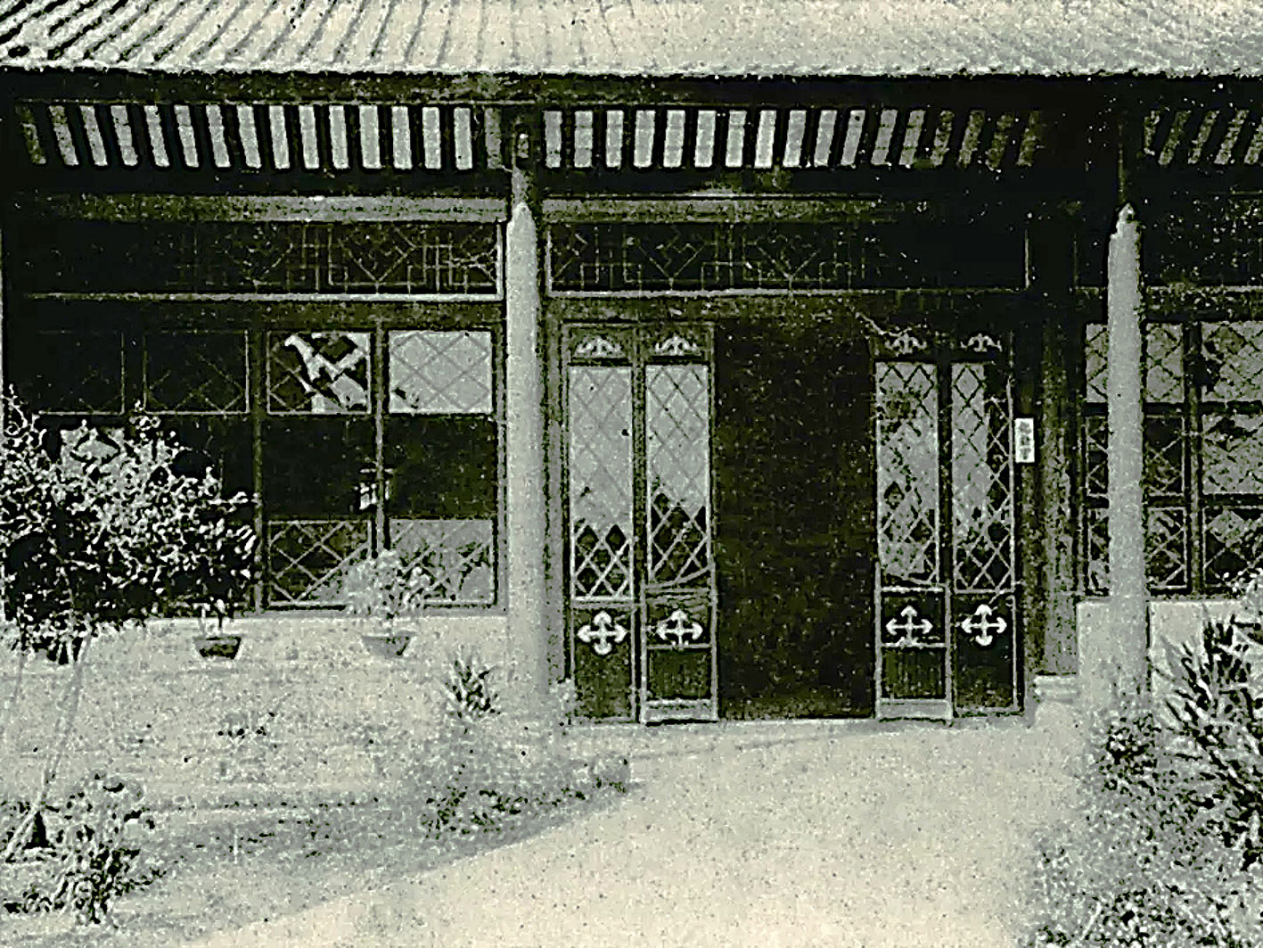
Guangdong Provincial Agricultural Testing Ground and Agricultural Workshop covered courses including the topics of general agriculture, soil, crops, farming tools, pests, etc

At the beginning of the second year of the Xuantong of the Qing Dynasty, after the Chinese Festival, classes at the testing site officially began. This project attracted a great deal of attention and the classes started to recruit students in February. One-thousand thirteen-hundred enthusiastic new students registered and enrolled. The courses on the testing site included the topics of general agriculture, soil, crops, farming tools, pests, sericulture, silkworm breeding methods, climatology, physics, and agronomy among other courses. Some of the teachers hired included Yin Li (利寅), who had returned to China from studies abroad in England, as well as Qianfu Guan (关乾甫) and Songshuo Chen (陈颂硕) who both returned from Japan (Fig. 4).

Around the time when the first students began registering to study at the site, the Qing Imperial Court issued an official communication approving the “Setup of Industrial Teachers College”, and then the Agricultural Institute began officially enrolling students. Given its strong faculty and comprehensive equipment, in March of the same year, the testing site began operations and changed its name to Agricultural Instructor Workshop. At this point in the school’s history, according to the “Characteristics of the Industrial Instructor’s Workshop”, the designation of “Higher Education School of Agriculture” was added to the teaching site. This was in keeping with Youheng Tang’s heart-felt deep commitment and purposed towards the expansion of agricultural education. Director Youheng Tang advocated for the combination of scientific research, education, and promotion of agriculture throughout his tenure as the leadership at Agricultural Institute, opening a new chapter of higher agricultural education in modern Guangdong.

**Figure 5:**
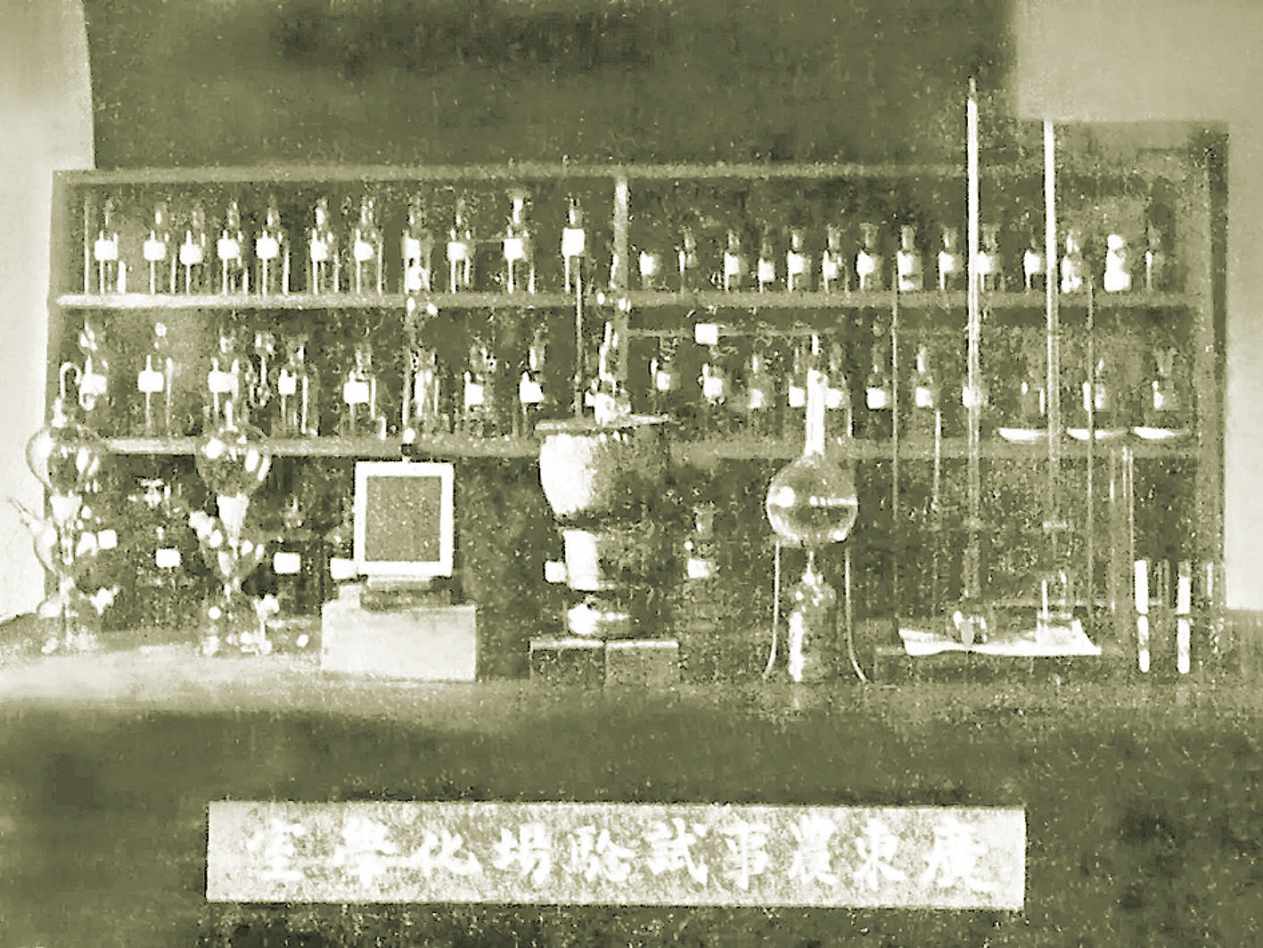
The chemistry room of Guangdong Provincial Agricultural Testing Ground

Youheng Tang leveraged the experience he learned abroad running an agricultural experimental school and knowledge of agronomy education to develop China’s rudimentary application of agricultural knowledge and address the pressing issue of talent shortage in the country. To resolve these issues, he creatively proposed new strategies to extend the semester to ensure and improve the quality of teaching. He personally formulated teaching plans and administrative rules and regulations. In addition to the original 13 basic agronomy courses, he added 23 new humanities courses to ensure greater comprehensive learning. These courses included morality, arithmetic and measurement, horticulture, veterinary medicine, aquaculture, agricultural manufacturing, and agricultural finance. Due to the tight course schedule at his institution, Youheng Tang observed that some first-year students had a poor foundation in natural sciences, and then arranged for the teachers to tutor these courses at night and on holidays to overcome this issue. In addition, he led the teachers to conduct a rigorous evaluation in order to check the knowledge and skill students gained at the institute. Most importantly, Youheng Tang insisted on the coexistence of lecture and practice, and the combination of practice and theory to promote each other (Fig. 5).

Youheng Tang made great achievements in agriculture and forestry education. He proposed that agricultural education should be rooted in cultivating modern agricultural talents in combination with promoting new science and agricultural laws. In the 12th year of the Republic of China (1923), Shizhao Zhang (章士钊), the first president of Beijing Agricultural University, hired Youheng Tang to be a professor, the director of the agronomy department, and a farming expert for the university. In the 17th year of the Republic of China (1928), while maintaining his professorship in Beijing, Youheng Tang also established an agricultural college at Anhui University. In the 23th year of the Republic of China (1934), he was appointed as the Secretary-General of Jinan University. In the 24th year of the Republic of China (1935), Youheng Tang served as the director and professor of the Department of Agronomy at Guangxi University. After the victory of the Second Sino-Japanese War in 1946, Youheng Tang served as the professor at Guangzhou University. During this period, he also held important positions at the agricultural colleges of other agriculture schools and higher education institutions in China, such as Tsinghua University.

As a pioneer, scientist, and educator, Youheng Tang who yearned for democracy, freedom, and progress, not only pioneered the modern agricultural education and scientific research in China, but also trained a batch of agricultural and forestry professionals for Guangdong province. In April 1912, upon graduation, the first-stage graduates of the Agricultural Science and Forestry Instructor’s Training Program were transferred to the members of the Ministry of Agriculture and Forestry of the Central Committee of the Republic of China. Just like their respected teacher, Dr. Tang, the recent alumni worked as teachers to promote the development of China’s agriculture, forestry, and agroforestry. Before the founding of the People’s Republic of China, relatives and friends advised Youheng Tang to settle in Taiwan and even booked a ticket for him, but he was unmoved and replied to his children and grandchildren: “Our generation should stick to our positions and welcome the changes that are a part of life.”

After the founding of the People’s Republic of China, Youheng Tang, despite his age and physical decline, actively served as a librarian at the Guangzhou Cultural and Historical Museum and the representative of the People’s Congress of Dongshan District, Guangzhou. Youheng Tang devoted himself to the compilation of agricultural history to express his gratitude to the Communist Party of China. In 1958, Youheng Tang died in Guangzhou at the age of 74. He made great contributions to the country where he full-heartedly dedicated his life to agricultural and agro-scientific research, greatly improving the nation’s agricultural education, pedagogy, farming technology, and farming methods.

## REFLECTING ON THE PAST, EXAMINING THE PRESENT, AND STRIDING INTO THE FUTURE

**Figure 6:**
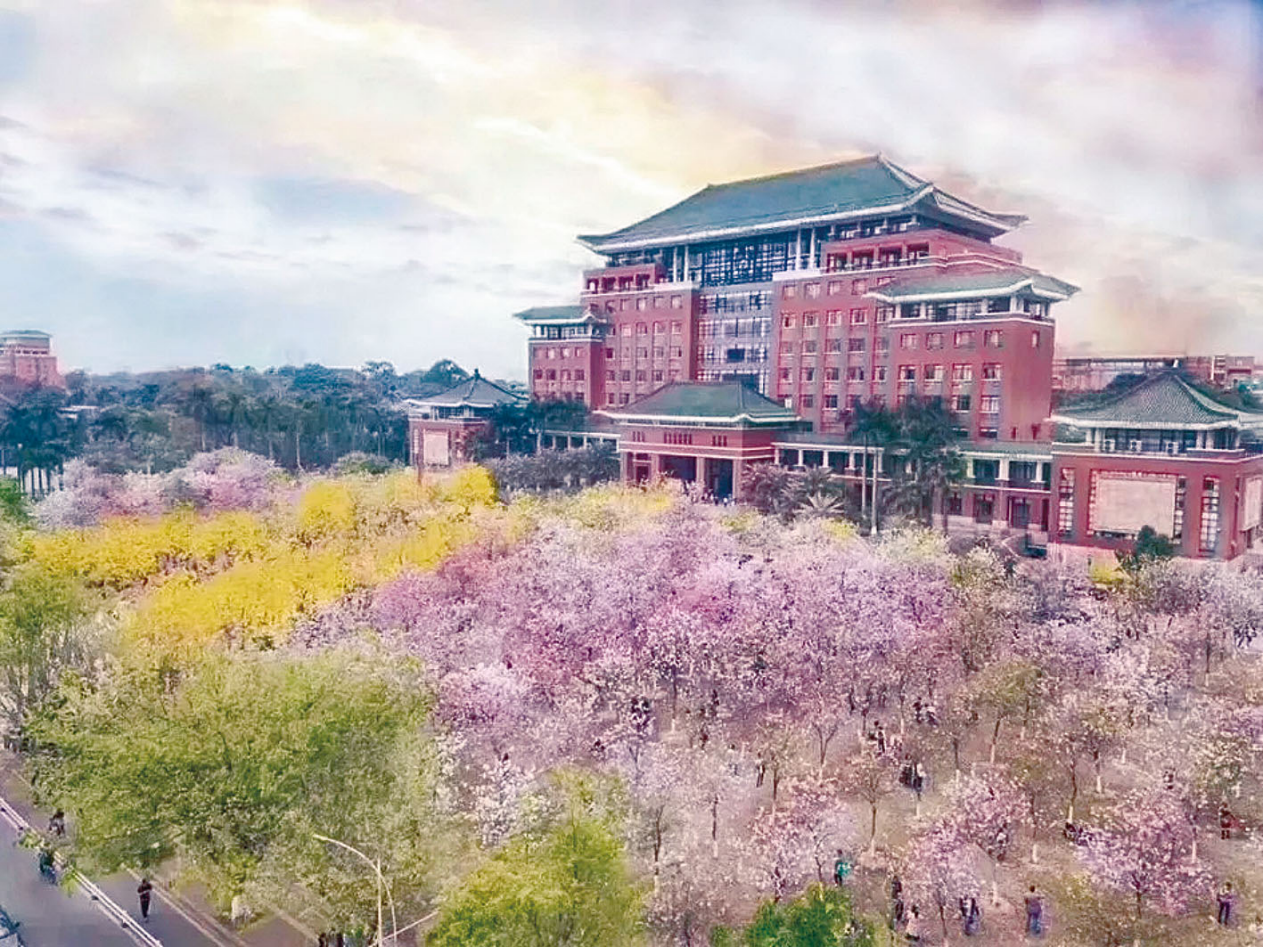
Campus with bauhinia flowers and the landmark of “five lakes, four grasses, and a forest”

As the years have passed, the world has changed dramatically. The former site, more than 100 years ago, of Guangdong Provincial Agricultural Testing Ground (now near Nonglin Road, Ouzhuang District in Guangzhou) has become a place with towering buildings of bright lights and bustling traffic. Researchers at South China Agricultural University still hold the same initial spirit and mission of their predecessors, and adhere to Youheng Tang’s belief in the 21st century. Anyone who has seen how our university grew from a mere institute with less than 100 students to the national university housing over 50,000 students today would say Youheng Tang’s efforts have come to fruition. Beginning from a small testing site in Dongshanshimagang to the present 8,270-acre campus with bauhinia flowers, which was called “five lakes, four lawns, and a forest”, South China Agricultural University today is a key national university with productive research activity and continues to expand and stride into the new research era. The numerous outstanding graduates from South China Agricultural University are the strongest proof of Youheng Tang’s efforts. In 2019, South China Agricultural University celebrated its 110th anniversary. Let us together eagerly anticipate the future of South China Agricultural University and hope that it will create brilliant achievements for China in the new area (Fig. 6).
